# Microstructure and Micro-Mechanical Properties of Thermally Sprayed HA-TiO_2_ Coating on Beta-Titanium Substrate

**DOI:** 10.3390/ma18030540

**Published:** 2025-01-24

**Authors:** Abdulaziz Kurdi, Doaa Almalki, Ahmed Degnah, Animesh Kumar Basak

**Affiliations:** 1 Advanced Materials Technology Institute, King Abdulaziz City for Science and Technology, P.O. Box 6086, Riyadh 11442, Saudi Arabia; akurdi@kacst.gov.sa; 2 King Salman Center for Disability Research, Riyadh 11614, Saudi Arabia; 3 Department of Pharmacy, Thadiq General Hospital, Third Health Cluster, Ministry of Health, Thadiq 15242, Saudi Arabia; doalmalki@moh.gov.sa; 4 Adelaide Microscopy, The University of Adelaide, Adelaide, SA 5005, Australia; 5 Centre for Research Impact & Outcome, Chitkara University Institute of Engineering and Technology, Chitkara University, Rajpura 140401, Punjab, India

**Keywords:** spark plasma sintering (SPS), TiO_2_-HA composite, microstructure, micro-pillar compression, disability

## Abstract

Metallic biomaterials in a solid form cause stress-shielding in orthopedic applications. Such implants also suffer from limited tissue attachment to become a part of the living system. In view of that, hydroxyapatite (HA) coating reinforced with titanium oxide (TiO_2_) was deposited in a beta (β)-Titanium (Ti-35Nb-7Ta-5Zr) substrate by plasma spray. This allows us to exploit the best of the two materials, namely the relatively low modulus of β-Ti, together with the porous and bone-like structure/composition of the HA to facilitate cell growth. This is foreseen to be used as an implant, particularly for musculoskeletal-related disability. Detailed scanning electron microscopy (SEM) investigation shows the lamellar structure of the coating that is composed of different phases and some porosities. Transmission electron microscopy (TEM) confirms the co-existence of both the amorphous and crystalline phases that build up the coating structure. In situ micro-mechanical tests revealed that the HA-TiO_2_ coating was low in strength and modules compared to that of the substrate material, together with lower ductility. The yield stress and modulus of elasticity of the coating were about 877 ± 174 MPa and 447 ± 24 MPa, respectively. In contrast, the beta (β)-Ti substrate possesses about 990 ± 85 MPa of yield stress and 259 ± 19 MPa modulus of elasticity. The deformation mechanism was also quite different, where the coating crumbled under compressive loading, featuring limited ductility with cleavage (brittle)-type fracture, and the substrate showed plastic flow of materials in the form of slip/shear planes with extended ductility.

## 1. Introduction

Metallic biomaterials are widely used for various orthopedic applications such as implants, and among them, the most common ones are stainless steel (particularly SS316L), Ti6Al4V, and Co-Cr [1,2,3]. The common issues related to metallic biomaterials as implants are threefold: (i) stress-shielding [2,4] due to high elastic modulus (about 110 GPa for Ti6Al4V) than bone (10–30 GPa) [2], (ii) release of toxic ions due to the corrosion of the implants in the body fluids [5], and (iii) low surface hardness and poor adhesion strength of metallic biomaterial surfaces (TiO_2_ layer in case of Ti6Al4V and Cr-oxide layer in case of stainless steel) with tissue, which deteriorates in the human body fluids over a period of time. For example, Ti6Al4V causes cytotoxicity and localizes irritation, which restricts the formation of tissue growth, and results in implant failure [6]. The same goes for stainless steel, which suffers from localized corrosion in the human body fluid, thus releasing harmful metallic ions in blood stream, degrading the strength of the implant over a period of time [7]. Moreover, bundles of collagenous white fibrous tissue form between Ti-based implant and bone, which leads to bone resorption and implant loosening issues [8]. Thus, withstanding excellent mechanical properties and biocompatibility, solid metallic biomaterials are unable to effectively comply with osseointegration criteria. Hence, an alternative to the well-known SS316L and Ti6Al4V biomaterial is aluminum and vanadium free beta (β)-type titanium (Ti). Recently, Ti-Nb-Ta-Zr type β-Ti-alloy has caught the attention of researchers for orthopedic applications as implants, particularly for musculoskeletal-related disability [9,10,11]. This type of β-Ti-alloy possesses low elastic modulus in the range of about 55 GPa, thus stress shielding can be avoided with better corrosion resistance and excellent biocompatibility [12,13].

Irrespective of the type, solid biomaterials suffer from limited interaction between the implants and tissue, which hampers efficient tissue binding. This is because of the non-active and low-surface-energy nature of the implants. One option to tackle such shortcomings is to modify the implant surface by incorporating a porous layer, which is bio-active in nature [14]. In these regards, surface modification by depositing hydroxyapatite (HA) coating has been widely exercised [15]. HA is a bio-ceramic, possesses the very same elemental composition as natural bone, and is the main inorganic component of bone. It has been established that HA can enhance the surface energy and bio-activity of metallic biomaterials [16]. Hydroxyapatite (HA) is widely used as a biomaterial coating, and exhibits improved bio-activity (cell proliferation), corrosion resistance, antibacterial properties, etc. The reason behind this is the open-pore structure of the HA coating, which allows the cells to grow in between the structure, and become part of the system with time. The inherent properties of the HA are low strength, elastic modulus, and fracture resistance due to the cracks, surface defects, and porosities in the coating structure [17,18,19].

To exploit the best of open-pore structure (e.g., HA-based coating) and solid biomaterials (e.g., bio-metallic alloys), HA-based coatings can be deposited on solid biomaterials. Under such circumstances, both the strength of the solid biomaterials and the bio-activity aspect of porous HA-based coating can be realized. On the other hand, the limited adhesion aspects of the solid biomaterial with bone tissues can also be overcome. Withstanding the excellent bio-activity of HA, it cannot be used as a standalone structure, as it possesses low-level fracture resistance, low bond strength with the substrate material, and moreover, it is brittle and fragile in nature [19]. Thus, it does not possess sufficient physio-mechanical properties to withstand long-term performance. Usually, HA is toughened by incorporating reinforcing particles, like TiO_2_ [20,21], ZrO_2_ [22], yttria-stabilized zirconia (YSZ) [23], Al_2_O_3_ [17], carbon nanotubes (CNTs) [24], graphene [25,26], and SiO_2_ [27] to enhance its physio-mechanical properties [28]. This is similar to that of reinforcing metal matrix with hard particles, like tungsten-carbide [29,30]. Nearly a 93% increase in adhesion strength was achieved in 60 wt. % TiO_2_-incorporated HA coating [27]. In addition to that, there was a substantial increase in cohesion by incorporating TiO_2_ in HA coating, and it was proven beneficial in reducing delamination [22]. This TiO_2_ incorporation in HA also reduces the wear rate from 0.6 mg·(N·m)^−1^ to 0.38 mg·(N·m)^−1^, where the reinforcement loading was 30 wt. % in the HA matrix, and increases the hardness from 1.65 GPa (without any reinforcement) to 2.95 GPa [31]. Moreover, HA-TiO_2_ coating exhibits better hydrophilic and biocompatible aspects than HA coating [31]. As reported by Prakash et al. [32], such HA-TiO_2_ coating promoted the proliferation of osteoblastic cells due to the formation of sub-micron scale craters, which provided excellent surface energy to promote cell growth equally on all surfaces and started proliferating at a high rate. Thus, it is not required to take the implants off after recovery. It can be seen that amongst various reinforcements, TiO_2_ has been most widely used to improve cohesive strength, abrasive wear resistance at micro-/nano-scale [33], hardness, and the bio-performances of coating [2]. Moreover, it has been reported that the reinforcement of TiO_2_ in HA significantly improved corrosion resistance [22]. Zheng et al. reported that the reinforcement of TiO_2_ to HA improved the adhesive strength, corrosion resistance, and bio-activity of HA coatings [34].

HA-based coatings can be applied via a wide range of techniques, such as micro-arc oxidation [35], sol–gel [36,37], electrolytic deposition [38,39], electrochemical anodic oxidation [40], sputtering ion coating [41], physical vapor deposition [42], electric discharge machining [14], plasma spraying [43,44], and ball-burnishing-assisted EDC process [32]. Among various coating deposition processes, plasma spray coating has been proven the best and ultimate solution for the deposition of HA-based coating on commercially available biomaterials [45]. According to the guidelines of the Food and Drug Administration (FDA), USA, the plasma spray technique is the potential candidate to deposit hydroxyapatite (HA)-based biomimetic coating on various bio-metallic materials [24,25,45].

Most of the related work towards HA-TiO_2_ coating deals with parametric optimization [46], microstructural morphology [14], corrosion, in vitro bio-activity [25], and limited mechanical property exploration, like hardness [46] and adhesion strength with the substrate [17]. However, there was no investigation related to the strength of the HA-TiO_2_ coating, which is of paramount importance to understanding the true mechanical behavior of such coatings. This was mostly due to the experimental difficulties, as the coating profile (thickness) lies in a couple of hundreds of micro-meters. This narrow structural profile does not allow us to fabricate ‘dog-bone’-shaped specimens for traditional macro-scale tensile testing. Thus, micro-pillar compression is the only way to evaluate the micro-mechanical properties of such coating. Having said that, it is a complementary technique towards the overall characterization of the coating structure and understanding the deformation behavior of such coatings under loading. This ‘knowledge gap’ was addressed in the present work, which stands as the novelty of this investigation. This experimental shortcoming was overcome by employing micro-scale testing procedures known as in situ micro-pillar compression [47]. Such micro-scale compression tests were successfully conducted on both coatings [48,49] and bulk materials [50,51] to unravel fundamental deformation behavior in addition to assessing the micro-scale mechanical properties.

Thus, the aim of the present work is to investigate the mechanical properties of the HA-TiO_2_ coating deposited by plasma spraying on metallic biomaterials on a micro-scale. Towards that, an in situ micro-pillar compression technique was employed. In addition, the deformation mechanism of such coating under compression, in the micro-scale, was also explored.

## 2. Materials and Methodology

### 2.1. Materials and Coating Deposition

As reported in the literature [32,46], the sole deposition of HA via thermal deposition leads to the decomposition of HA and forms the amorphous structure, which inherently possesses detrimental mechanical properties. This was overcome by adding TiO_2_ in HA, which not only reduced the brittleness, but also improved ductility and elastic modulus [52]. These second-phase particles act as a reinforcing medium in the coating structure and enhance the load-bearing capacity of the coating.

The plasma deposition technique was used to deposit the coating on a β-Ti substrate with the dimension of 10 cm × 10 cm × 5 mm. Before the coating deposition, the substrate was sand-blasted for better anchoring of the coating. After that, the surface was cleaned with ethanol to remove the loose grits and grease/oil. High purity (>98%) HA powder (10–25 μm) and TiO_2_ powder (20–50 μm) were procured from Sigma Aldrich, St. Louis, MO, USA, and mixed together in a ratio of 70:30, and used as a feedstock for coating deposition by atmospheric plasma spraying. The plasma spraying parameters are given in Table 1, which were selected based on the recommendations from the literature to obtain a dense coating structure with optimized properties [53].

### 2.2. Coating Characterization Technique

The deposited coating was characterized as follows: “After coating deposition, the specimen was sectioned with a water-cooled diamond saw to carry out hot resin mounting and metallographic polishing. The polishing was conducted in Struers automatic polishing machine (Ballerup, Denmark), with varying degree of diamond slurry, and final polishing with colloidal silica suspension. The specimen was prepared in a cross-sectional direction, and the microstructure was investigated by field emission scanning electron microscope (Quanta 450 FE-SEM, Thermofisher Scientific, Waltham, MA, USA), and Oxford energy dispersive X-ray (EDX) analysis system (Oxford Instruments, Abingdon, UK). Transmission electron microscopy (TEM) was carried out with a probe corrected Titan TEM (Thermofisher Scientific, USA) at 200 KV. Both micro-pillars and TEM samples were prepared by a focused ion beam (FIB-SEM) (Helios Nanolab 600, Thermofisher Scientific, USA)”. Proper care was taken to prepare the cross-section of the samples, and it was ensured that no artifacts of sample preparation were on the obtained results.

### 2.3. Micro-Pillar Fabrication and Stress–Strain Calculation

A brief description of the micro-pillars fabrication and compression was given as follows: “A focused ion beam (FIB-SEM) system (Helios Nanolab 600, FEI, Hillsboro, OR, USA) was used to fabricate micro-pillars with Ø 3 µm. The micro-pillars were fabricated at the middle of 30 µm pit to avoid any contact of the indenter with the surrounding rim of the pit. Rough milling was carried out at 6.5 nA current at 30 kV, with final milling at 0.46 nA at 30 kV. A flat diamond punch of Ø 5 µm mounted on in situ nanoindentation system (PI 88, Hysitron, MN, USA) was used to conduct the compression tests. During compression, a 3 nm/s loading rate was selected, which corresponds to a 10^−3^ s^−1^ strain rate. The unloading rate was 50 nm/s. The whole process was recorded via videos. The automated computerized system recorded the load-displacement curves during micro-pillar compression. The data collected were converted into stress-strain curves, according to the equations, as stated below with due references. Deformed micro-pillars were also investigated by SEM.

Applied normal force (*F*) and corresponding change of pillar length (*Δl*) were recorded during compression by a computer-controlled program, and subsequently used to calculate stress, according to Equation (1):

(1) $$\sigma =\frac{F}{A_{0}}$$

where *σ* is stress, *F* is normal force and *A*_0_ is the cross-sectional area of the pillar at 25% of its height from the top. As the pillars were slightly taper (<2°), thus, the most probable deformation will happen closer to the top surface [54].

Total displacement (*U_total_*) during loading contains two parts: change of the pillar length *Δl* and displacement induced from Sneddon’s effects, *U_Sneddon_*, which includes elastic penetration of the pillar. It can be expressed as [25,26]:

(2) $$u_{total}=∆l+u_{Sneddon}$$

(3) $$u_{Sneddon}=\frac{(1-\nu^{2})P}{2E}\sqrt{\frac{\pi }{A_{s}}}$$

where, *ν* and *E* are Poisson’s ratio and Young’s modulus, correspondingly; *P* is applied load, *A_s_* is cross-sectional area of pillar base. By replacing Equations (2) and (3), the change of length, *Δl*, could be computed as:

(4) $$\Delta l=u_{total}-\frac{(1-\nu^{2})P}{2E}\sqrt{\frac{\pi }{A_{s}}}$$

Thus, average engineering strain $\varepsilon_{E}$ can be calculated as:

(5) $$\varepsilon_{E}=\frac{∆l}{l_{0}}$$

where $\varepsilon_{E}$ is strain, *Δl* is change of pillar length, and *l*_0_ is initial pillar length. Details of the equations can be found in literature [55]. A number of micro-pillars were fabricated to ensure reproducibility, and at least five individual experiments were conducted in each case”.

## 3. Results and Discussion

### 3.1. Microstructural Characterization

**Figure 1:**
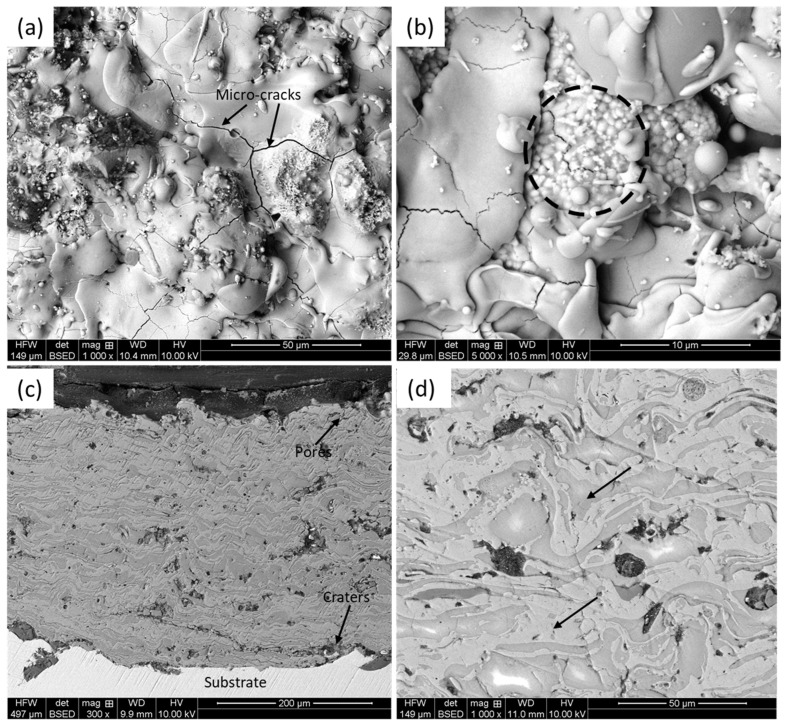
Back-scattered SEM micrographs on HA-TiO_2_ coating: (**a**,**b**) planner view of as-received coating (without metallographic polishing) and (**c**,**d**) cross-sectional view (after metallographic polishing).

A representative microstructure of the HA-TiO_2_ coating, in terms of back-scattered electron image, on both the planner and cross-sectional directions, is shown in Figure 1. From the planner view (Figure 1a,b) of the coating (without metallographic polishing), it was confirmed that there were many micro-cracks (point with arrows in Figure 1a), spherical particles, partially melted particles, and agglomerates (points out with dotted circle in Figure 1b). Thus, the coating possesses a heterogeneous surface appearance with partially and fully melted splats [32]. The agglomeration (pointed out by the dotted circle in Figure 1b) occurred due to the difference in the melting point of HA (1500 °C) and TiO_2_ (1800 °C), together with the disintegration of the HA into Ca_3_(PO_4_)_2_ and CaO phases, during deposition, as reported by Yao et al. [22]. The cross-section of the coating confirmed that it was about 400 μm thick with a dense and compact morphology. The characteristic lamellar-type microstructure was evident (Figure 1c), which formed as the oncoming splats of molten/semi-molten droplets of material stuck on top of each other, and built up the coating thickness. The usual defects, such as micro-pores, craters, and partial/unmelted particles, were unevenly distributed. This was an inherent nature of the plasma-sprayed HA-TiO_2_ coating, as reported by other researchers [32,53]. Having said that, such defects in the coating structure were not detrimental for the present sought-out applications, as such the pores and crates “acted as a reservoir to hold the protein in the host body and improved the osseointegration and corrosion resistance” [24].

**Figure 2:**
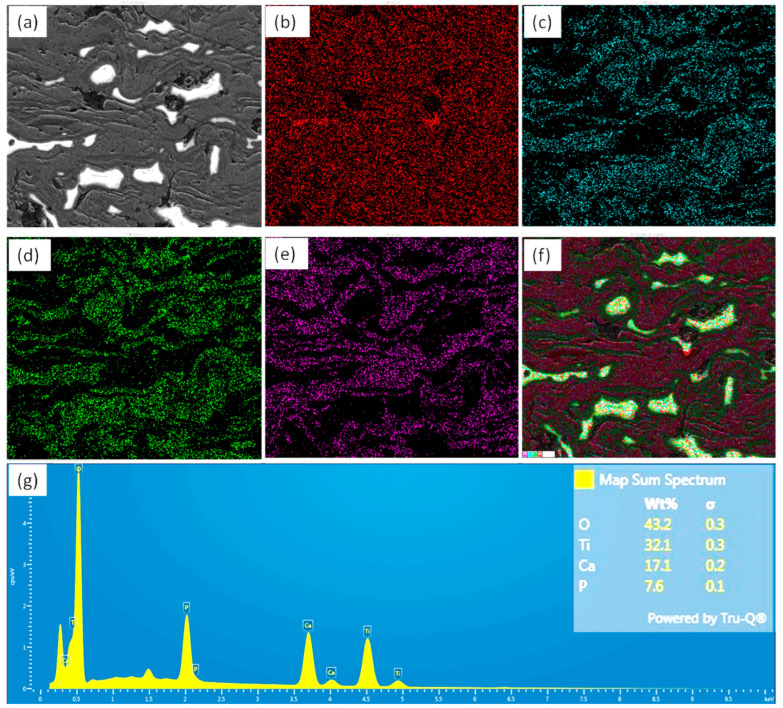
Elemental mapping on the cross-section of HA-TiO_2_ coating: (**a**) secondary electron image, (**b**) O map, (**c**) Ca map, (**d**) P map, (**e**) Ti map, (**f**) EDS layered image, and (**g**) map sum spectrum.

The elemental mapping of the coating (on cross-section), together with the corresponding spectrum, is shown in Figure 2. During elemental mapping, it was ensured that the sample block was placed flat, the detector inserted, and the SEM was used at 10 KV, with an aperture spot size of three to acquire sufficient counts. Figure 2a depicts the SEM image from where the corresponding elemental mapping of oxygen (Figure 2b), calcium (Figure 2c), phosphorus (Figure 2d), and Ti (Figure 2e) were collected. Figure 2f shows the layered image with the EDS spectrum in Figure 2g. As reported in the literature, during the thermal spraying process, the HA disintegrated into non-apatite phases, and formed α/β tricalcium phosphate (TCP), CaO, and tetracalcium phosphate (TTCP), along with TiO_2_ [56], which are bio-active in nature and improved the corrosion resistance and bio-performance of the coating [57]. From the EDX spectrum, it can be observed that the ratio of Ca and P was 2.25, which indicates the presence of tricalcium phosphate (TCP) and tetracalcium phosphate (TTCP) [20]. According to Heimann et al. [56], a Ca/P ratio of ≤1.67 indicated the formation of the amorphous calcium phosphate phase (ACP). In the present case, the Ca/P ratio was found as 2.25, which clearly indicated that the HA was not completely decomposed to the ACP phase.

**Figure 3:**
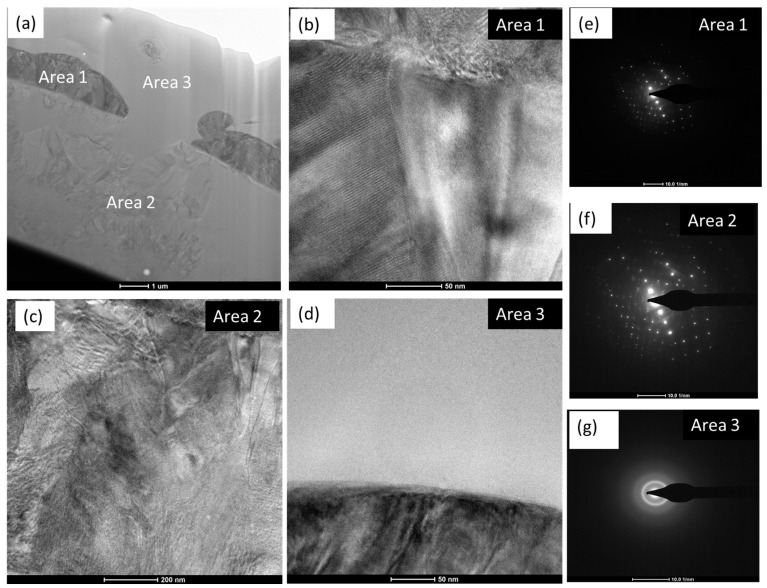
TEM micrographs on HA-TiO_2_ coating: (**a**) overall view of the coating representing different areas, (**b**–**d**) high resolution (HR)-TEM images of areas 1–3, respectively, as marked in (**a**) with corresponding electron diffraction pattern (**e**–**g**).

The microstructure of the coating was further investigated by TEM. Towards that, TEM foils were prepared by FIB-SEM, as stated in the experimental Section 2.2. Figure 3 depicts the outcomes of the TEM investigation, which comprise bright field TEM images at different locations (Figure 3a–d), along with selected area diffraction patterns in those areas (Figure 3e–g). Figure 3a represents the overall coating structure, which includes a darker region (area 1), semi-transparent region (area 2), and featureless region (area 3). The high-resolution images on those areas confirm the crystalline nature of area 1 (Figure 3b) and area 2 (Figure 3c), whereas area 3 (Figure 3d) appears as amorphous. This was further confirmed by the electron diffraction patterns, as shown in Figure 3e–g, respectively, for areas 1–3. The weak diffraction rings in area 1 (Figure 3e) and area 2 (Figure 3f) are apparently related to β-TCP, as well as the reflection spots of the <110> zone of TTCP, together with the crystalline structure of TiO_2_. Area 3 was totally amorphous, as confirmed by the hollow diffuse rings in Figure 3g. Thus, both the crystalline and amorphous phases co-exist in the coating microstructure. The formation of the amorphous calcium phosphate phase is due to the thermal decomposition of HA in the course of coating deposition. Ji et al. [58] and Li et al. [59] also reported the presence of such tricalcium phosphate (Ca_3_PO_4_)_2_ and calcium titanate (Ca_2_Ti_2_O_5_) phases that formed due to the result of the reaction between hydroxyapatite and titanium oxide (TiO_2_) in the plasma flame. In addition, sub-micron-sized equiaxed hydroxyapatite grains were apparent in area 1 (Figure 3a).

### 3.2. Compression of Micro-Pillars

**Figure 4:**
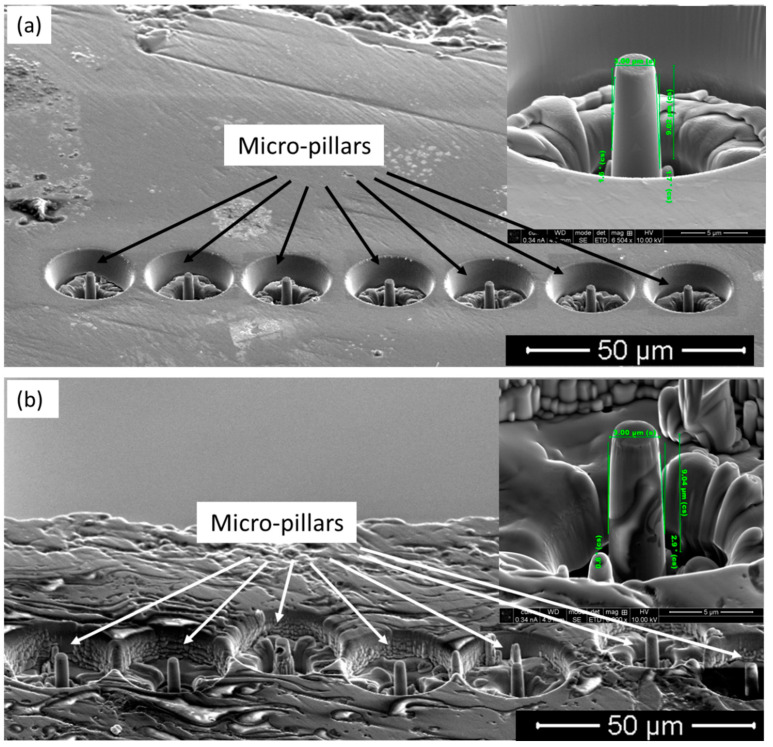
FIB-prepared micro-pillars for compression (**a**) on the substrate (β-Ti) and (**b**) on the HA-TiO_2_ coating. Enlarged views of the micro-pillars are shown as inserts in respective images.

As outlined in the experimental section, the micro-pillars were fabricated by Ga focused ion beam (FIB) milling on the cross-section of the coating, as well as on the substrate. To ensure the reproducibility of the results, a number of micro-pillars were fabricated in each case, as shown in Figure 4.

Figure 4a shows the micro-pillars on the substrate, with a high-magnification view of one of the micro-pillars as an insert, and the same goes for the micro-pillars on the coating, as shown in Figure 4b. The insert images confirm that the micro-pillars are homogeneous, with 3 μm in diameter and 9 μm in height; thus, a 1:3 aspect ratio was maintained to evade bucking during compression [60]. In the case of the coating, the micro-pillars (insert in Figure 4b) contained several phases, which was representative of the coating microstructure, as reported in Section 3.1.

**Figure 5:**
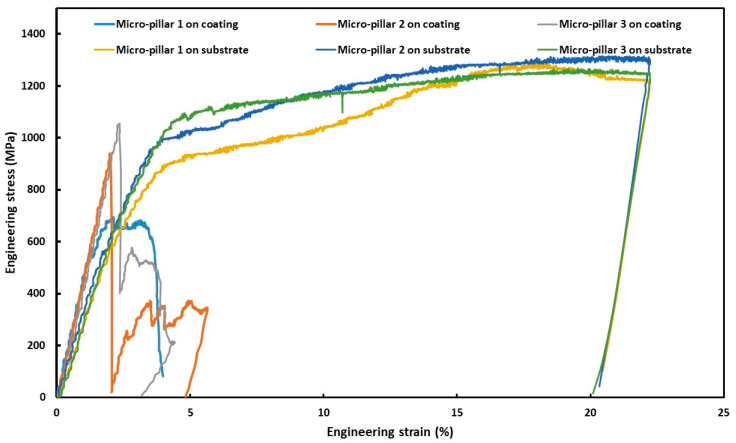
Stress–strain curves on the cross-section of the HA-TiO_2_ coating and substrate β-Ti metallic biomaterial subjected to micro-pillar compression.

During in situ compression, load was applied at a pre-set rate, and the corresponding change in displacement was digitally recorded by the instrument software. These raw data were processed according to the equations, as stated in Section 2.2, into stress–strain data and plotted accordingly, as shown in Figure 5.

Three representative graphs on each sample are presented in Figure 5, though at least seven samples were compressed in each case for the neatness of the graph and ease of comparison. However, all the data were used to calculate the average values and standard deviation, as reported in Table 2.

As shown in Figure 5, there was a striking difference between the micro-mechanical behaviors of these two materials. First of all, both the stress and strain accommodated by the coating were much lower than that of the substrate. Once the coating experienced yield stress, there was no further accommodation of strain, as the micro-pillars collapsed within about 2% of the strain. On the other hand, the substrate exhibited extended plasticity (strain accommodation) representative of metallic materials [61]. Thus, it can be stated that the coating was prone to break down under limited strain, which was not completely unexpected, as evident in the literature in terms of their hardness values. As reported by Singh et al. [17], the hardness of the HA-TiO_2_ coating increases with the increase in TiO_2_ content, and the optimum reinforcement content was reported as 30 wt. %, which depicts a surface hardness of 2.95 GPa compared to 1.65 GPa for sole HA coating deposited by plasma spraying [31]. The elastic modulus of as-sprayed HA and HA-TiO_2_ (30 wt. %) coatings were measured as 15.7 GPa, and 41.75 GPa, respectively. Prakash et al. [32] reported the hardness of HA-TiO_2_ (30 wt. %) as 848 HV, obtained by ball-burnishing-assisted electric discharge cladding possess, which was significantly higher (285 HV) than that of the substrate (β-Ti alloy) [32]. In addition to hardness, the reinforcement also increased the adhesion strength up to 32.5 MPa in comparison to 18.5 MPa for the sole HA coating [53]. The elastic modulus of as-sprayed HA and HA-30 wt. % coatings were measured at 15.7 GPa and 41.75 GPa, respectively [53]. It was not possible to make any direct comparison of the micro-mechanical properties of the presently investigated coating with the literature, as this was the first report on that. In the literature, only hardness and elastic modulus derived from hardness were reported.

### 3.3. Deformation Under Compression

**Figure 6:**
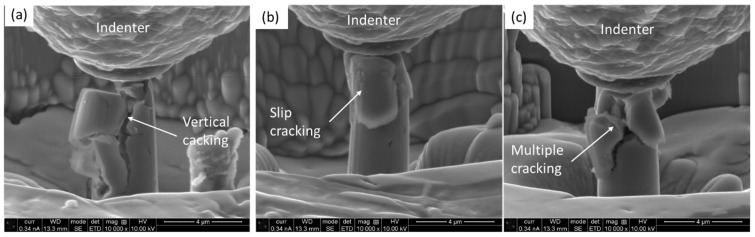
Deformed micro-pillars on the coating cross-section after compression: (**a**) micro-pilalr 1, (**b**) micro-pillar 2 and (**c**) micro-pillar 3.

As reported in the experimental section, the compression was conducted in situ, which enabled us to capture every moment of the process via secondary electron (SE) image video recording in high resolution. Some moments of the process on three different micro-pillars on the costing are depicted in Figure 6. It was evident, irrespective of the given micro-pillars, that severe cracking and deformation took place, which led to the micro-pillar failure.

**Figure 7:**
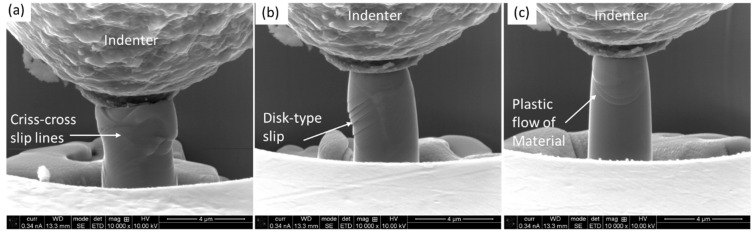
Deformed micro-pillars on the β-Ti substrate after compression: (**a**) micro-pilalr 1, (**b**) micro-pillar 2 and (**c**) micro-pillar 3.

In Figure 6a, the micro-pillar experiences vertical cracking, where a portion of the micro-pillar is still hanging under the indenter. Figure 6b shows that a portion of the micro-pillar chipped off as a result of cracking. Similarly, Figure 6c depicts the multiple cracking and crumbling of the pieces. In contrast to that, the micro-pillars on the substrate held as a single piece even after experiencing extended strain, as shown in Figure 7. Irrespective of a given micro-pillar on the substrate, the deformation mode was the propagation of the slip and shear planes, as marked out in Figure 7a–c. The relative movements of the slip/shear planes caused the formation of disk-type protruded materials and plastic flow. All the micro-pillars remained intact until the end of the compression.

**Figure 8:**
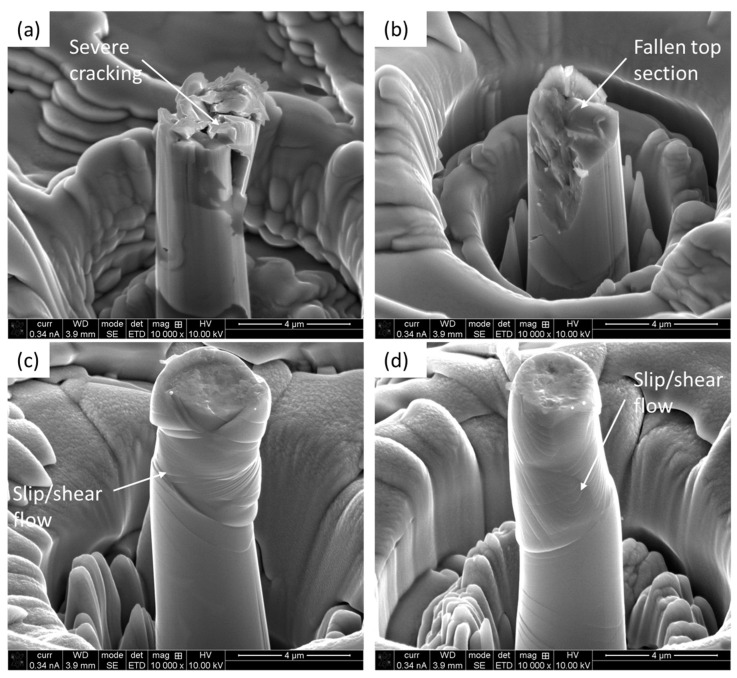
Deformed micro-pillars on coating (**a**,**b**) and substrate (**c**,**d**) after compression.

The deformed micro-pillars were further investigated after the completion of compression, and the representative images of two micro-pillars in each case are shown in Figure 8. In the micro-pillars on the coating, extensive cracking took place (Figure 8a), which made it fragmented, whereas in one case, the top of the micro-pillar completely fell (Figure 8b). As stated earlier, the thermally sprayed coating showed characteristics of a lamellar microstructure in the presence of splat boundaries. In addition to that, during the thermal spraying process, the individual splats get solidified almost immediately. All of these lamellar structures of the coating and the existence of mixed crystalline–amorphous phases were likely to facilitate crack nucleation and propagation under compression. As evident from the stress–strain graphs (Figure 5), the coating exhibits limited plastic flow compared to the substrate due to the absence of slip/shear plane formation and propagation. In the case of the micro-pillars on the substrate, it withstands numerous slip and shear bands. These slip/shear bands do not follow any specific orientation, though the preferred orientation is 30–45°, as reported in the literature [62], due to the occurrence of critically resolved shear stresses in that range [63].

Thus, it can be summarized that the HA-TiO_2_ coating showed much lower ultimate compressive strength compared to the β-Ti substrate. This was not unexpected, given the nature of the coating itself. Having said that, the ultimate objective of these coatings was not the load-bearing application, but to facilitate the bone regeneration process, and became a part of the musculoskeletal system when used as an implant to address physiological disability due to osteoporosis. As reported in the literature [64], in contrast to HA coating, HA-TiO_2_ coating possesses higher adhesion strength because of two reasons: (i) the reinforcement of TiO_2_ particles, and (ii) phase purity and content. As reported by Balani et al. [24], such reinforcement not only enhances the load-bearing capacity of the coating to a limited extent, but also improves the wear resistance and decreases the friction in contact.

## 4. Conclusions

In this work, biomimetic HA coating loaded with 30 wt.% TiO_2_ was successfully deposited on a β-Ti substrate by using a plasma spray deposition technique. The HA-TiO_2_ coating exhibited a dense, lamellar appearance, with about 400 μm in thickness. By considering the observed results, the present study unequivocally advocates the potential use of HA-TiO_2_ for orthopedic applications. The major conclusions of the present study were as follows:

1. The plasma spraying deposition of the HA-TiO_2_ coating gives rise to a lamellar-like structure, which incorporates different phases, together with minimal structural defects, such as porosities. The as-deposited surface also contains solidification cracks and the agglomeration of unmelted TiO_2_ coated with HA.
2. The strength and accommodated strain of the coating (877 ± 174 MPa of yield strength and <2% of strain) was considerably lower than that of the substrate (990 ± 85 MPa of yield strength and >20% of strain). This was due to the very physical nature of the coating itself. In contrast, the modulus of elasticity of the coating (447 ± 24 MPa) was about 1.72 higher than the substrate (259 ± 19 MPa).
3. The presence of splats, together with crystalline and amorphous phases causes cleavage-type brittle fracture on the coating under compressive loading with minimal ductility. In contrast, the deformation mechanism of the substrate comprises the propagation of slip and shear planes with considerable ductility, characteristic of metallic materials.

The micro-mechanical properties of thermally sprayed HA-TiO_2_ coating exhibit a promising aspect for the broad biomedical relevance of such coatings. One of the limitations noticed of such coating in the present research was the brittleness under compression. The addition of 30 wt. % TiO_2_ into HA was not enough to subdue the brittleness of such coatings. Future work may consider the incorporation of nanoparticles, which may enhance the cohesion within the coating structure, and thus reduce the brittleness further. In addition, the effect of hybrid fillers may also be explored.

## Figures and Tables

**Table 1:** Plasma spray process parameters [31].

Spraying Parameters	Value
Primary gas flow (Argon)	6.41 × 10^−4^ m^3^/s
Secondary gas flow (Hydrogen)	3.33 × 10^−5^ m^3^/s
Carrier gas flow (Argon)	8.33 × 10^−5^ m^3^/s
Voltage	64 volt
Current	500 Amp
Feed rate	32 gm/min
Spray distance	120 mm

**Table 2:** Micro-mechanical properties of the HA-TiO_2_ coating in cross-section and substrate β-Ti metallic biomaterials calculated from the stress–strain curves. * as the pillars collapsed just after the yield point, just yield strength was considered as ultimate compressive strength.

Investigated Material	Yield Strength (σ_y_), MPa	Ultimate Compressive Strength (σ_UCS_), MPa	Elastic Modulus (E), MPa
Substrate (β-Ti)	990 ± 85	1269 ± 21	259 ± 19
HA-TiO_2_ coating	877 ± 174	877 ± 174 *	447 ± 24

## Data Availability

The raw/processed data used to produce the results will be made available by the corresponding author upon reasonable request due to privacy.
